# Supplementary material for: Cetacean coronavirus spikes highlight S glycoprotein structural plasticity
Source: PLoS Pathog. 2026 Apr 8;22(4):e1013855. doi: 10.1371/journal.ppat.1013855 (PMC13061240; doi:10.1371/journal.ppat.1013855)
Supplement: S1 File — a) Anti-strep Western blot and SDS-PAGE of CeCoV spike protein ectodomains used for cryoEM. b) Negative stain micrographs of BwCoV and BdCoV spike ectodomains used for structural characterization. Micrographs display monodisperse particles that are of the expected size (~10 x 20 nm). Size bar represents 100 nm. Fig B. Cryo-EM data processing pipelines in cryoSPARC10 for BwCoV (left panel) and BdCoV S (right panel). Fig C. Cryo-EM data processing of the BwCoV (left) and BdCoV (right) spike ectodomains. a) FSC plots of BwCoV and BdCoV spike ectodomain reconstructions. b) Representative reference-free 2D class averages. c) Tightly contoured EM density maps for the spike ectodomains coloured according to local resolution which was calculated in cryoSPARC10 (countour level BwCoV: 0.156; BdCoV: 0.0544). d) Loosely contoured EM density maps for the spike ectodomains coloured according to local resolution which was calculated in cryoSPARC10 (countour level BwCoV: 0.0931; BdCoV: 0.0206). Part of the flexible S1A loop decorated with O-glycans at the apex of the trimer can be observed at this contour level (dashed black boxes). e) Angular distribution plot calculated in cryoSPARC10 for particle projections in the globally refined map. f) Examples of EM density + model to illustrate resolution of maps. Top panels: BwCoV, bottom panels BdCoV. Panels left to right: S1A, S1B, S1C, S2. Fig D. CeCoV S quaternary packing is reminiscent of alphaCoV spike proteins. Surface representations are shown of representative spike proteins from all four genera (IBV (6cv0), PDCoV (6b7n), SARS-CoV-2 (6vxx), HCoV-NL63 (5szs). Protomers of the trimeric models are coloured in the same manner for every protein. Models are ordered according to their packing mode: spike proteins displaying primarily inter-protomer contacts (beta-, gammaCoV) on the left and spike proteins displaying primarily intra-protomer contacts (alpha-, deltaCoV) on the right. The BwCoV spike structure is highlighted by a dashed re [file ppat.1013855.s001.pdf]

Cetacean coronavirus spikes highlight S glycoprotein structural plasticity

Ruben J.G. Hulswit[a#], Tatiana M. Shamorkina[b], Joline van der Lee[a], Floor Rosman[a], Lisanne S. Wetzels[a], Frank J.M. van Kuppeveld[a], Joost Snijder[b], Berend Jan Bosch[a#], Daniel L. Hurdiss[a#]

[a]Virology Section, Infectious Diseases and Immunology Division, Department of Biomolecular Health Sciences, Faculty of Veterinary Medicine, Utrecht University, Utrecht, the Netherlands.

[b]Biomolecular Mass Spectrometry & Proteomics, Bijvoet Center for Biomolecular Research, Department of Chemistry, Faculty of Science, Utrecht University, Utrecht, the Netherlands.

[#]Corresponding authors: Ruben J. G. Hulswit, r.j.g.Hulswit@uu.nl; Daniel L. Hurdiss, d.l.hurdiss@uu.nl and Berend-Jan Bosch, b.j.bosch@uu.nl

Cetacean coronavirus spikes highlight S glycoprotein structural plasticity – **Supplementary Information**

Table of Contents S1 Appendix

Figure A Purification of BwCoV and BdCoV SED-GCN4-ST (trunc3) 2P proteins and negative stain data.

Figure B Cryo-EM data processing pipelines for BwCoV and BdCoV S.

Figure C Cryo-EM data processing of the BwCoV and BdCoV spike ectodomains

Figure D CeCoV S quaternary packing is reminiscent of alphaCoV spike proteins.

Figure E FoldTree analysis of coronavirus $S1^B$ domains supports ancestral relation of CeCoV $S1^B$ to alpha- and deltacoronaviruses.

Figure F AlphaFold 3 structure prediction models of the BdCoV and BwCoV $S1^0$ domains does not reflect experimentally determined structures.

Figure G CeCoV S displays distinctive elements in conserved S2 fusion machinery.

Figure H Detection of N-glycans on the surface of CeCoV spike glycoprotein.

Figure I Detection of O-glycans on the surface of the CeCoV spike glycoprotein.

Figure J Additional glycosylation data for CeCoV S proteins.

Table A Cryo-EM data collection, refinement and validation statistics for global refinements.

Table B Overview of unresolved regions in CeCoV N-terminal domains

Table C Overview of experimentally detected N-glycan abundance on coronavirus spike proteins

Table D Overview and conservation of predicted N-linked glycosites across CeCoV BwCoV and BdCoV S proteins along with the corresponding experimental data.

Data S1 BwCoV_AUC_per_glycosite_all_proteases.xlsx

Data S2 BdCoV_AUC_per_glycosite_all_proteases.xlsx

Data S3 N_glycol_site_detection_per_protease.xlsx

Data S4 O_glycans_Opair_pooledData.xlsx

References

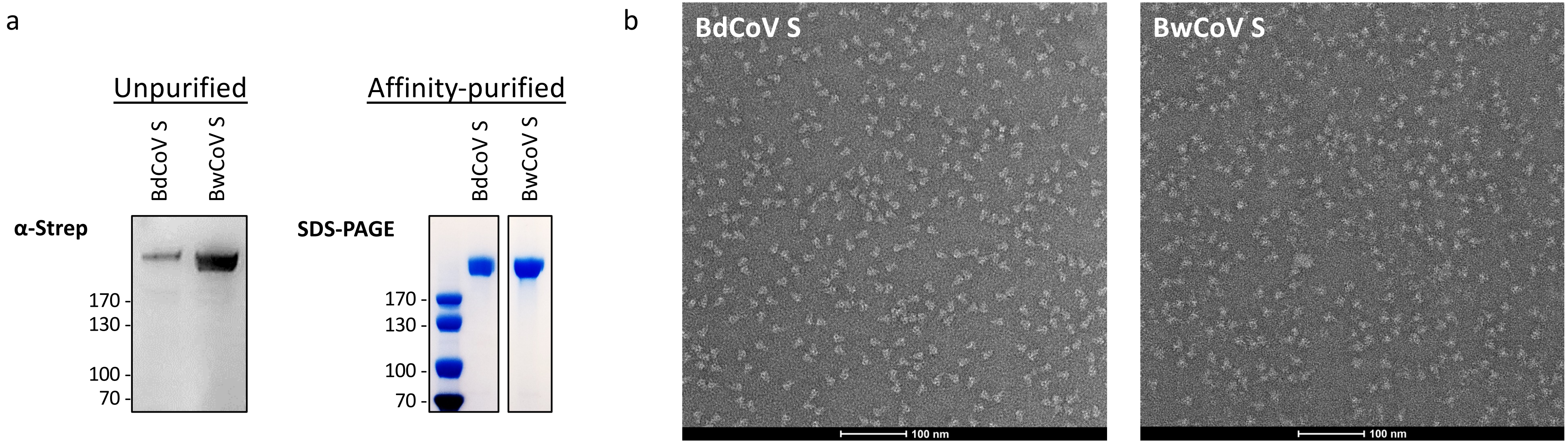

Figure A Purification of BwCoV and BdCoV SED-GCN4-ST 2P proteins and corresponding negative stain data. a) Anti-strep Western blot and SDS-PAGE of CeCoV spike protein ectodomains used for cryoEM. b) Negative stain micrographs of BwCoV and BdCoV spike ectodomains used for structural characterization. Micrographs display monodisperse particles that are of the expected size (~10 x 20 nm). Size bar represents 100 nm.

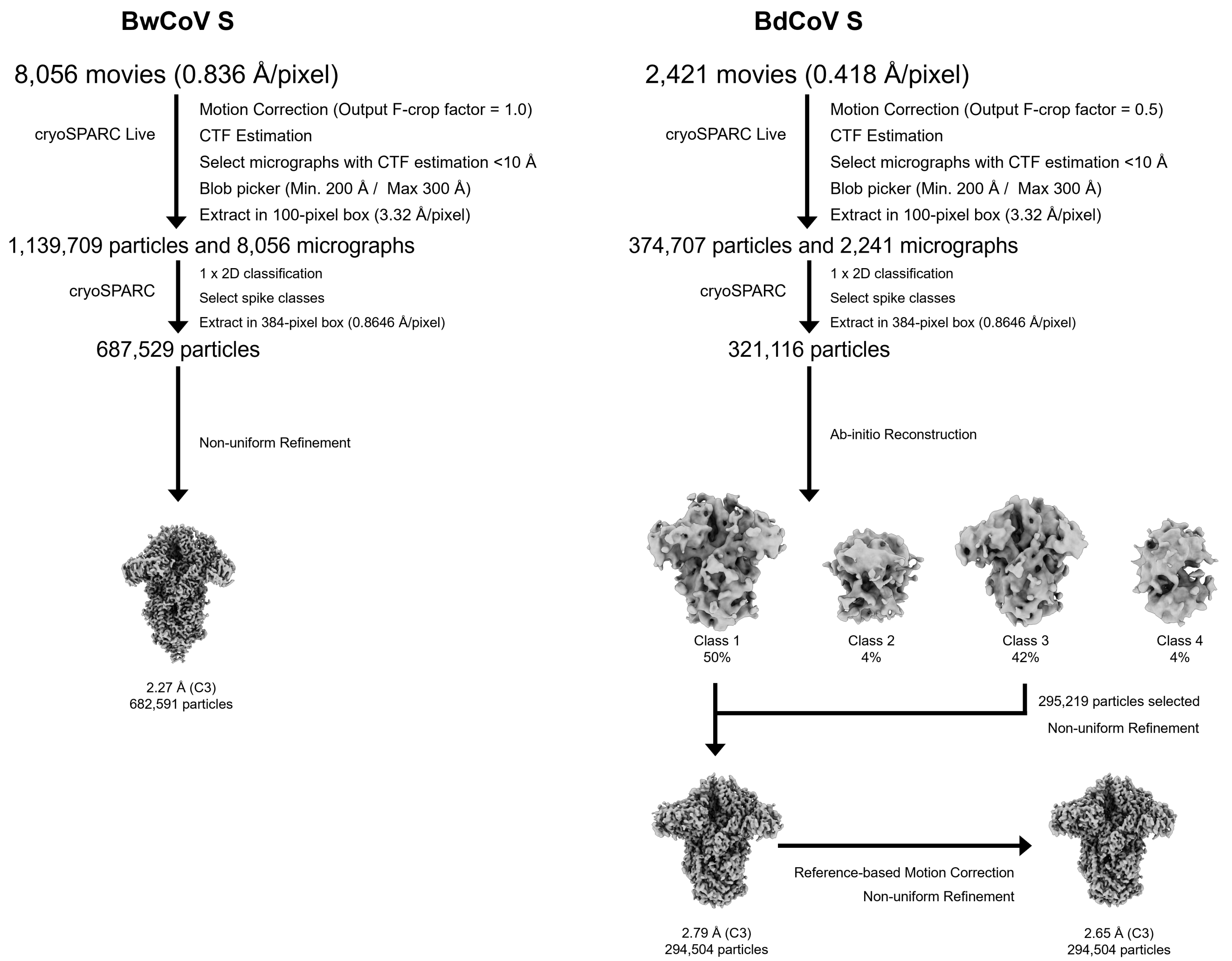

Figure B Cryo-EM data processing pipelines in cryoSPARC[10] for BwCoV (*left panel*) **and BdCoV S** (*right panel*).

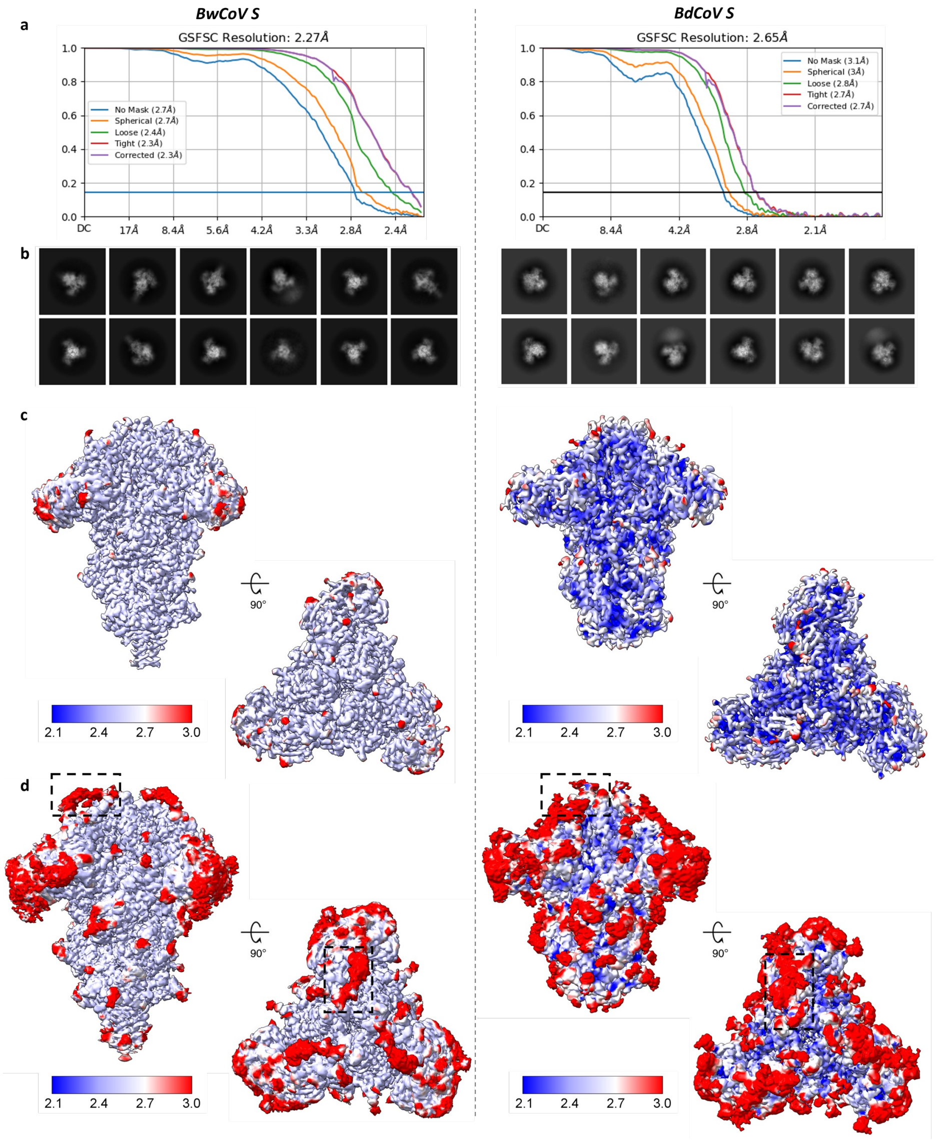

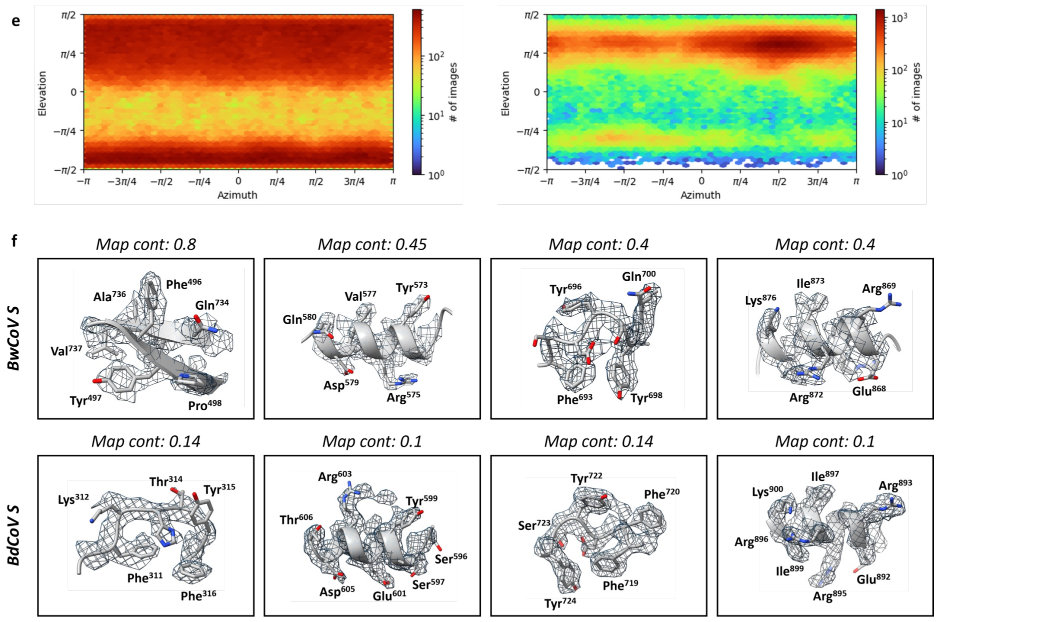

Figure C Cryo-EM data processing of the BwCoV *(left)* **and BdCoV** *(right)* **spike ectodomains.** a) FSC plots of BwCoV and BdCoV spike ectodomain reconstructions. b) Representative reference-free 2D class averages. c) Tightly contoured EM density maps for the spike ectodomains coloured according to local resolution which was calculated in cryoSPARC[10] (countour level BwCoV: 0.156; BdCoV: 0.0544). d) Loosely contoured EM density maps for the spike ectodomains coloured according to local resolution which was calculated in cryoSPARC[10] (countour level BwCoV: 0.0931; BdCoV: 0.0206). Part of the flexible S1^{A} loop decorated with O-glycans at the apex of the trimer can be observed at this contour level (dashed black boxes). e) Angular distribution plot calculated in cryoSPARC[10] for particle projections in the globally refined map. f) Examples of EM density + model to illustrate resolution of maps. Top panels: BwCoV, bottom panels BdCoV. Panels left to right: S1^{A}, S1^{B}, S1^{C}, S2.

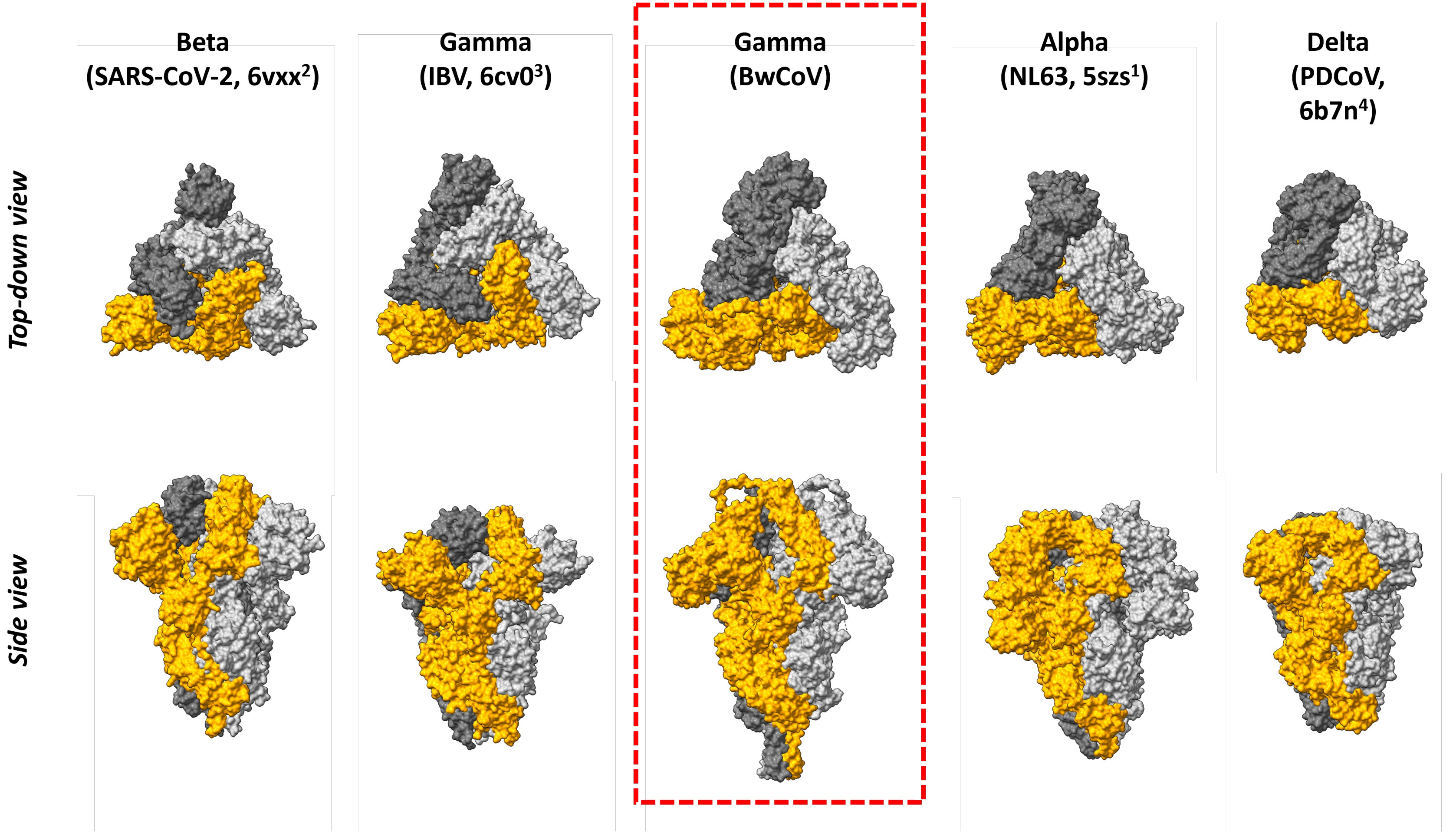

Figure D CeCoV S quaternary packing is reminiscent of alphaCoV spike proteins. Surface representations are shown of representative spike proteins from all four genera (IBV (6cv0), PDCoV (6b7n), SARS-CoV-2 (6vxx), HCoV-NL63 (5szs). Protomers of the trimeric models are coloured in the same manner for every protein. Models are ordered according to their packing mode: spike proteins displaying primarily inter-protomer contacts (beta-, gammaCoV) on the left and spike proteins displaying primarily intra-protomer contacts (alpha-, deltaCoV) on the right. The BwCoV spike structure is highlighted by a dashed red box.

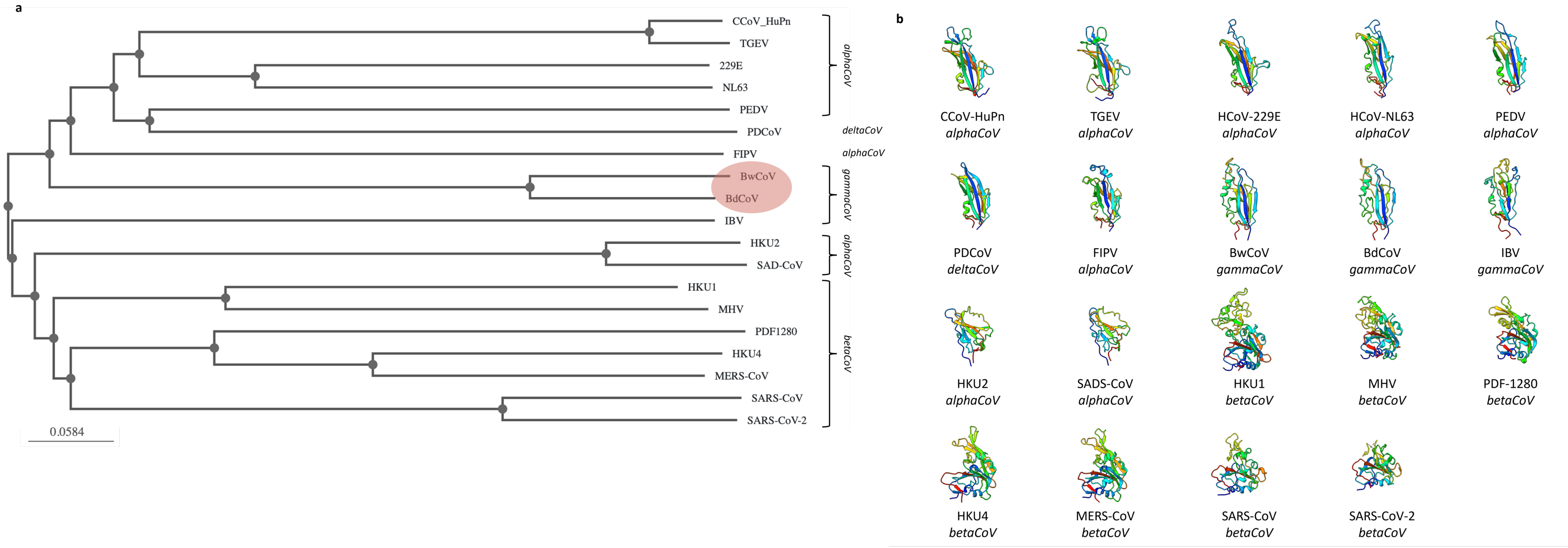

Figure E FoldTree[11] analysis of coronavirus $S1^B$ domains supports ancestral relation of CeCoV $S1^B$ to alpha- and deltacoronaviruses. A) FoldTree analysis of experimentally characterized coronavirus $S1^B$ domains. B) Cartoon representation of aligned and rainbow-coloured atomic models of experimentally characterized coronavirus $S1^B$ domains. The models are shown in the order of the phylogeny for comparison.

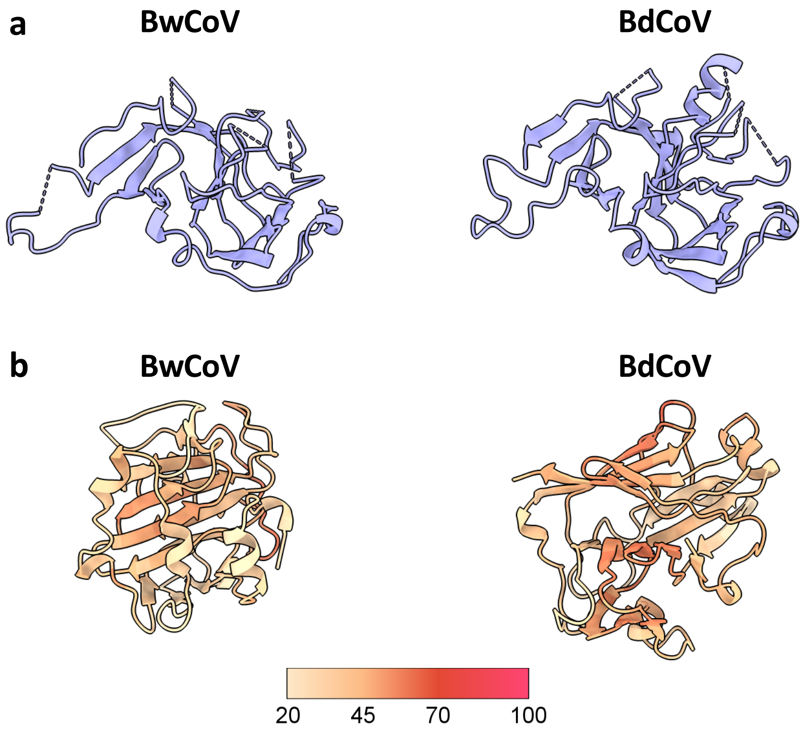

Figure F. AlphaFold 3 structure prediction models of the BdCoV and BwCoV S1⁰ domains does not reflect experimentally determined structures. a) Cartoon representation of the experimentally determined structures of BdCoV and BwCoV $S1^0$, shown in the same (aligned) view. Unresolved regions are indicated by dashed lines, no glycans are shown for clarity. b) Cartoon representation of the top AlphaFold 3[14] models predicted for the amino acid sequences of domain $S1^0$ of the BdCoV and BwCoV spike proteins. Models were aligned to the experimentally determined structures shown in panel a and are coloured by pIDDT scores. The colour legend indicates the pIDDT confidence level (a higher score signifies a higher confidence).

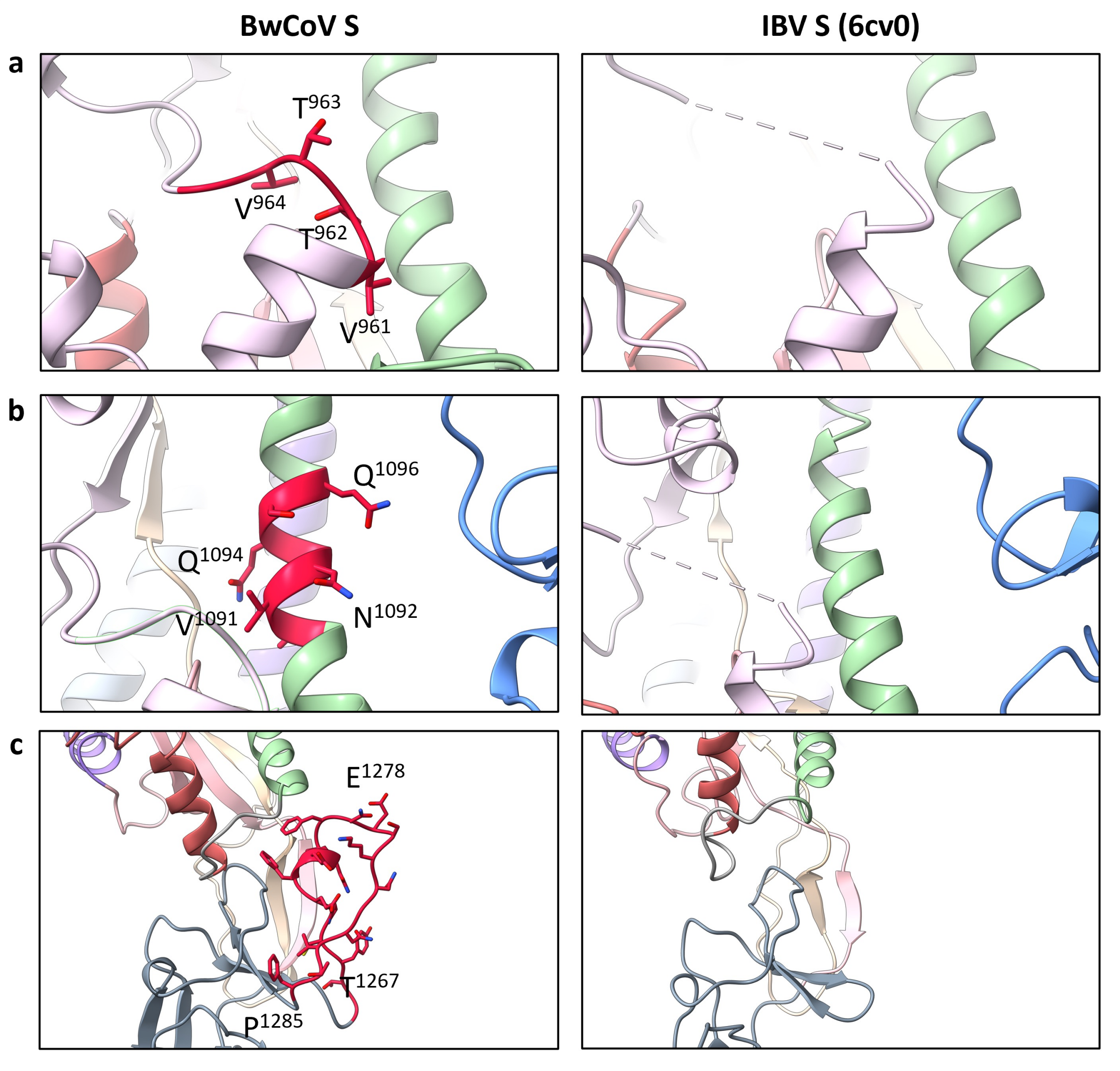

Figure G CeCoV S displays distinctive elements in conserved S2 fusion machinery. a) Cartoon representation of the S2 stalk region of BwCoV (*left panel*) and IBV S (*right panel*) with the different S2 elements coloured as in Fig 1c for reference. The 5-residue insertion directly upstream of S2' site found in CeCoV S proteins (BwCoV residues Ser961 – Asp965; BdCoV residues Ser984 – Gly988) is shown in as red sticks coloured by heteroatom. b) The insertion of one heptad repeat in HR1 (BwCoV residues Thr1090 – Val1096; BdCoV residues Thr1113 – Val1119) is shown as red sticks coloured by heteroatom. c) The 21-residue insertion in HR2 (BwCoV residues Gly1266 – Phe1286; BdCoV residues Gly1289 – Phe1309) is shown as red sticks coloured by heteroatom.

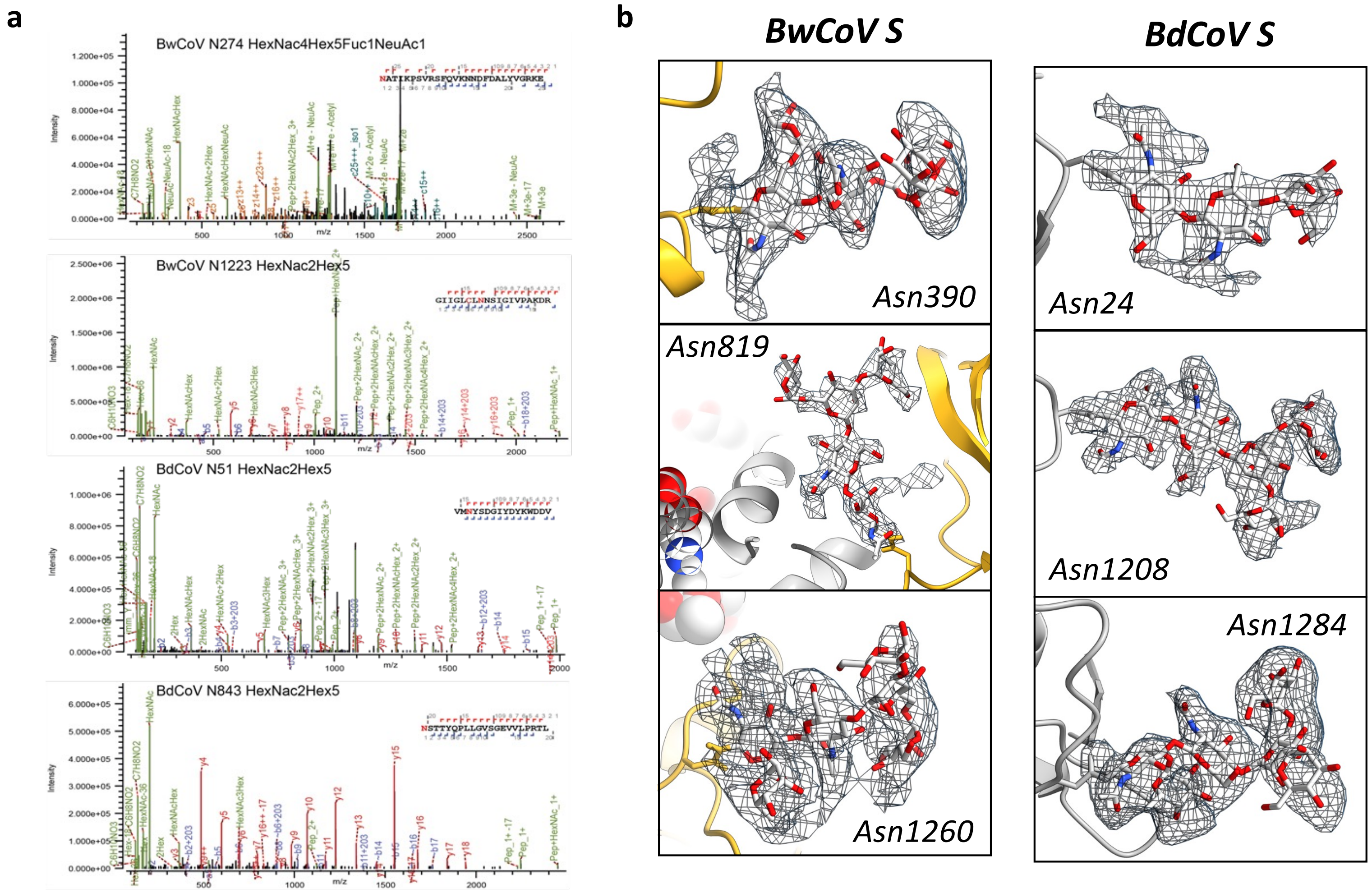

Figure H Detection of N-glycans on the surface of CeCoV spike glycoprotein. a) Representative MS/MS spectra of the Byonic N-glycan identifications for BwCoV (top two panels) and BdCoV (lower two panels). b) EM density (grey mesh) zoned around the indicated modelled N-glycans of the BwCoV (*left panels*) and BdCoV (*right panels*) spike glycoproteins.

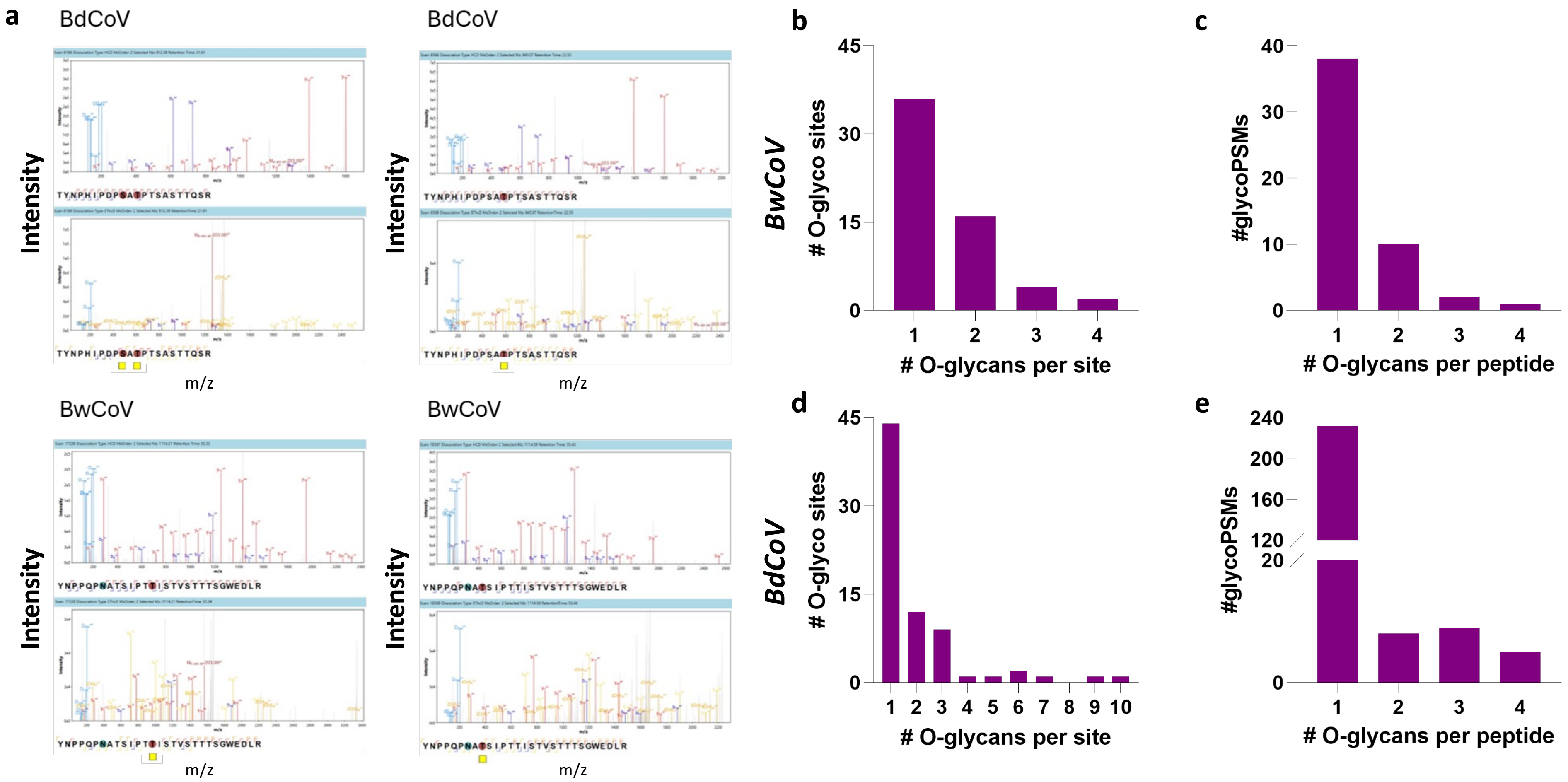

Figure I Detection of O-glycans on the surface of the CeCoV spike glycoprotein. a) Representative MS/MS spectra supporting Opair identifications of O-glycosylation sites. Top spectra show HCD fragmentation (b- and y-ions; blue and red, respectively) and identification of oxonium ions (light blue); bottom spectra show EThcD fragmentation where additionally c- and z-ions can be seen (yellow and orange, respectively). b) and d) Number of identified O-glycosites with the number of identified O-glycoforms per site for BwCoV and BdCoV S, respectively. Most identified O-glycosites carry only one glycoforms per site. c) and e) Number of O-glycans identified on the same peptide.

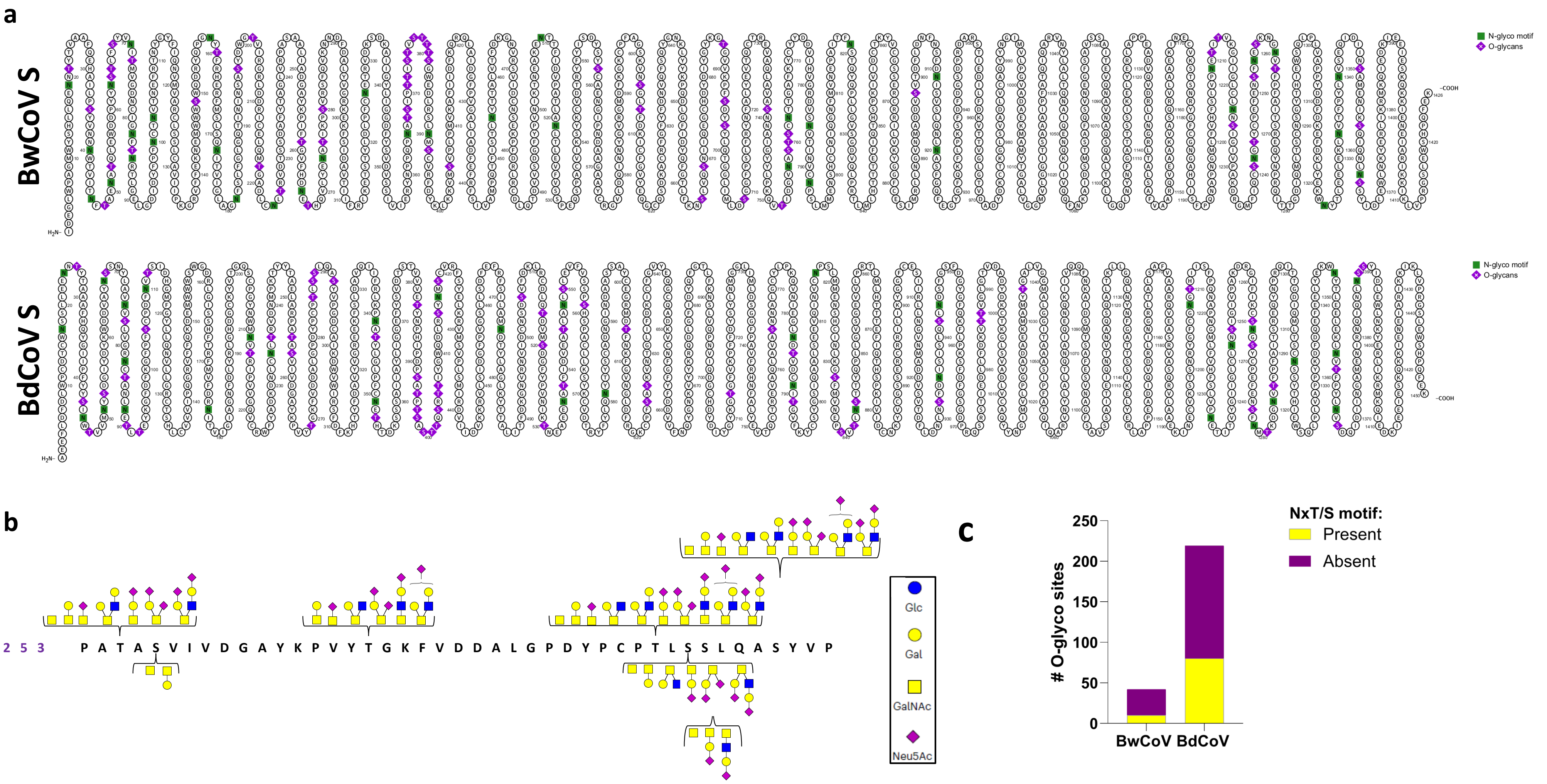

Figure J Additional glycosylation data for CeCoV S proteins. a) Overview of experimentally detected N- and O-glycosylation patterns of both CeCoV S proteins. N-glycan sites are depicted in green and O-glycan sites in purple. b) Overview of dominant *O*-linked glycoforms within BdCoV residues 379-410. c) Number of O-glycosylation sites identified within an N-glycan motif for both CeCoV S proteins.

Table A Cryo-EM data collection, refinement and validation statistics for global refinements.

	BwCoV S (EMDB-53512) (PDB 9R1Q)	BdCoV S (EMDB-53513) (PDB 9R1R)
**Data collection and processing**		
Magnification	105,000 x	105,000x
Voltage (kV)	300	300
Electron exposure (e-/Å²)	50	50
Defocus range (μm)	-	-
Pixel size (Å)	0.83	0.83
Symmetry imposed	C3	C3
Initial particle images (no.)	1,139,709	374,707
Final particle images (no.)	682,591	294,504
Map resolution (Å)	2.27	2.65
FSC threshold	0.143	0.143
Map resolution range (Å)	2.18 - 20.69	1.81 - 15.95
**Refinement**		
Model resolution (Å)		
FSC threshold 0.5	2.30	2.68
FSC threshold 0.143	2.23	2.53
Map sharpening *B* factor (Å²)	41.82	54.08
**Model composition**		
Non-hydrogen atoms	38619	31422
Protein residues	3777	3663
Ligands	219	198
**B factors (Å²)**		
Protein	135.66	114.22
Ligand	130.61	119.10
**R.m.s. deviations**		
Bond lengths (Å)	0.005	0.005
Bond angles (°)	0.75	0.69
**Validation**		
MolProbity score	1.95	1.74
Clashscore	14.50	9.02
Poor rotamers (%)	0.00	0.28
**Ramachandran plot**		
Favoured (%)	97.26	96.14
Allowed (%)	2.65	3.53
Disallowed (%)	0.08	0.33

Table B Overview of unresolved regions in CeCoV N-terminal domains. Topologically equivalent regions of the two spike proteins are listed within the same row.

Domain	BwCoV spike	BdCoV spike
$S1^0$	57-63	
	85-96	91-101
	106-115	112-114
	151-162	160-165
	189-196	193-204
$S1^A$		226-230
	265-276	277-303
		392-414
S2	776-784 (around S1/S2 junction)	801-812 (around S1/S2 junction)
	1332-end	1333-end

Table C Overview of experimentally detected N-glycan abundance on coronavirus spike proteins. N-glycosylation calculated as 2.5 kDa addition to MW of glycoprotein.

Glycoprotein	N-glycosylation Sequons (kDa)	# protein residues (kDa)	N-glycan contribution to total MW	Reference
**HIV-1 gp**	31 (77.5)	859 (95)	44.9%	Kwon, 2015, *NSMB*[5]
**BwCoV S**	37 (92.5)	1449 (160)	36.6%	*This study*
**HCoV-NL63 S**	34 (85)	1356 (149)	36.2%	Walls, 2016, *NSMB*[1]
**BdCoV S**	35 (87.5)	1472 (162)	35.4%	*This study*
**LASV gp**	11 (27.5)	491 (54)	33.7%	Hastie, 2017, *Science*[6]
**HCoV-HKU1 S**	29 (72.5)	1356 (149)	32.6%	Watanabe, 2020, *Nat Comm*[7]
**SARS-CoV S**	23 (57.5)	1255 (138)	29.1%	Watanabe, 2020, *Nat Comm*[7]
**SARS-CoV-2 S**	22 (55)	1273 (140)	28.2%	Watanabe, 2020, *Science*[8]
**MERS-CoV**	23 (57.5)	1353 (149)	27.7%	Watanabe, 2020, *Nat Comm*[7]
**IAV HA (H1)**	9 (22.5)	566 (62)	26.6%	Lee, 2014, *Nat Comm*[9]

Table D Overview and conservation of predicted N-linked glycosites across CeCoV BdCoV and BwCoV S proteins along with their corresponding experimental data.

	BwCoV S				BdCoV S			
	Sequon	*Glycan detected by MS?*	*Protein region resolved in EM?*	*EM density for glycan chain?*	*Sequon*	*Glycan detected by MS?*	*Protein region resolved in EM?*	*EM density for glycan chain?*
1					17 – NSS	complex	Yes	Yes
2	19 – NNT	mannose	Yes	Yes	24 – NNT	mannose	Yes	Yes
3	40 – NWT	mannose	Yes	Yes	45 – NWT	mannose	Yes	Yes
4	46 – NFT	mannose	Yes	Yes	51 – NYS	mannose	Yes	Yes
	51 – NAT	complex	Yes	No				
5	63 – NST	complex	No	No	67 – NSS	complex	Yes	No (only low res)
6	71 – NIT	mannose	Yes	Yes	75 – NVS	mannose	Yes	Yes
7	82 – NFT	mannose	Yes	Yes	82 – NCT	mannose	Yes	Yes
8	85 – NRT	~none	No	No	88 – NET	complex	Yes	Yes
	100 – NCT	mannose	Yes	Yes				
9	103 – NVT	~none	Yes	No	110 – NVT	complex	Yes	No (only low res)
	158 – NYT	complex	No	No				
10	172 – NIT	complex	Yes	Yes	176 – NIT	mannose	Yes	Yes
	181 – NFS	mannose	Yes	Yes				
11					211 – NVT	mannose	Yes	Yes
12	224 – NLT	complex	Yes	Yes	234 – NLT	complex	Yes	Yes
	265 – NET	complex	No	No				
	274 – NAT	complex	No	No				
13					341 – NAT	mannose	Yes	Yes
14	326 – NET	complex	Yes	Yes	351 – NET	complex	Yes	Yes
	365 – NAT	mannose	Yes	Yes				
15	390 – NMS	complex	Yes	Yes	417 – NMS	complex	Yes	Yes
16					474 – NDT	mannose	Yes	Yes
	455 – NYT	mannose	Yes	Yes				
17	509 – NTT	complex	Yes	Yes	536 – NAT	complex	Yes	Yes
18	521 – NLT	hybrid	Yes	Yes	548 – NLS	mannose	Yes	Yes
19					581 – NYT	complex	Yes	Yes
20	757 – NAS	complex	Yes	Yes	783 – NDT	mannose	Yes	Yes
21	763 – NTT	complex/mannose	Yes	Yes	789 – NIT	complex	Yes	Yes
	785 – NVT	hybrid	Yes	No				
22	819 – NST	mannose	Yes	Yes	843 – NST	mannose/hybrid	Yes	Yes
23	911 – NIS	hybrid	Yes	Yes	935 – NIS	complex	Yes	Yes
24	920 – NAS	complex	Yes	Yes	944 – NDS	complex	Yes	Yes
25					1208 – NGT	mannose	Yes	Yes
26	1209 – NET	mannose	Yes	Yes	1233 – NET	mannose	Yes	Yes
27	1223 – NNS	complex	Yes	Yes	1247 – NNS	mannose	Yes	Yes
28	1242 – NGT	complex	Yes	Yes	1266 – NGS	mannose	Yes	Yes
29	1254 – NES	mannose	Yes	Yes	1278 – NMT	mannose	Yes	Yes
30	1260 – NVT	complex	Yes	Yes	1284 – NVT	complex	Yes	Yes
31					1314 – NSS	mannose	Yes	Yes
32	1323 – NYT	complex	Yes	Yes	1347 – NYT	~none	No	no
33	1333 – NVT	none	No	No	1357 – NVT	mannose	No	no
34	1340 – NVS	none	No	No	1364 – NVS	~none	No	no
35	1363 – NSS	complex	No	No	1387 – NSS	~none	No	no

Cetacean coronavirus spikes highlight S glycoprotein structural plasticity – **Supplementary Information**

References

1. Walls, A.C., Tortorici, M.A., Frenz, B., Snijder, J., Li, W., Rey, F.A., DiMaio, F., Bosch, B.-J., and Veesler, D. (2016). Glycan shield and epitope masking of a coronavirus spike protein observed by cryo-electron microscopy. Nat. Struct. Mol. Biol. *23*, 899–905. https://doi.org/10.1038/nsmb.3293.
2. Walls, A.C., Park, Y.J., Tortorici, M.A., Wall, A., McGuire, A.T., and Veesler, D. (2020). Structure, Function, and Antigenicity of the SARS-CoV-2 Spike Glycoprotein. Cell *181*, 281-292.e6. https://doi.org/10.1016/j.cell.2020.02.058.
3. Shang, J., Zheng, Y., Yang, Y., Liu, C., Geng, Q., and Luo, C. (2018). Cryo-EM structure of infectious bronchitis coronavirus spike protein reveals structural and functional evolution of coronavirus spike proteins. PLoS Pathog. *14*, e1007009.
4. Shang, J., Zheng, Y., Yang, Y., Liu, C., Geng, Q., Tai, W., Du, L., Zhou, Y., Zhang, W., and Li, F. (2018). Cryo-Electron Microscopy Structure of Porcine Deltacoronavirus Spike Protein in the Prefusion State. J. Virol. *92*, e01556-17.
5. Do Kwon, Y., Pancera, M., Acharya, P., Georgiev, I.S., Crooks, E.T., Gorman, J., Joyce, M.G., Guttman, M., Ma, X., Narpala, S., et al. (2015). Crystal structure, conformational fixation and entry-related interactions of mature ligand-free HIV-1 Env. Nat. Struct. Mol. Biol. *22*, 522–531. https://doi.org/10.1038/NSMB.3051.
6. Hastie, K.M., Zandonatti, M.A., Kleinfelter, L.M., Heinrich, M.L., Rowland, M.M., Chandran, K., Branco, L.M., Robinson, J.E., Garry, R.F., and Saphire, E.O. (2017). Structural basis for antibody-mediated neutralization of Lassa virus. Science (80-.). *356*, 923–928.
7. Watanabe, Y., Berndsen, Z.T., Raghwani, J., Seabright, G.E., Allen, J.D., Pybus, O.G., McLellan, J.S., Wilson, I.A., Bowden, T.A., Ward, A.B., et al. (2020). Vulnerabilities in coronavirus glycan shields despite extensive glycosylation. Nat. Commun. *11*, 2688. https://doi.org/10.1038/s41467-020-16567-0.
8. Watanabe, Y., Allen, J.D., Wrapp, D., McLellan, J.S., and Crispin, M. (2020). Site-specific glycan analysis of the SARS-CoV-2 spike. Science *9983*, eabb9983. https://doi.org/10.1126/science.abb9983.
9. Lee, P.S., Ohshima, N., Stanfield, R.L., Yu, W., Iba, Y., Okuno, Y., Kurosawa, Y., and Wilson, I.A. (2014). Receptor mimicry by antibody F045-092 facilitates universal binding to the H3 subtype of influenza virus. Nat. Commun. *5*. https://doi.org/10.1038/NCOMMS4614.
10. Punjani, A., Rubinstein, J.L., Fleet, D.J., and Brubaker, M.A. (2017). CryoSPARC: Algorithms for rapid unsupervised cryo-EM structure determination. Nat. Methods *14*, 290–296. https://doi.org/10.1038/nmeth.4169.
11. Moi, D., Bernard, C., Steinegger, M., Nevers, Y., Langleib, M., and Dessimoz, C. (2023). Structural phylogenetics unravels the evolutionary diversification of communication systems in gram-positive bacteria and their viruses. bioRxiv, 2023.09.19.558401. https://doi.org/10.1101/2023.09.19.558401.
12. Sievers, F., and Higgins, D.G. (2014). Clustal omega. Curr. Protoc. Bioinforma. 48, 3.13.1-3.13.16. https://doi.org/10.1002/0471250953.BI0313S48.
13. Robert, X., and Gouet, P. (2014). Deciphering key features in protein structures with the new ENDscript server. Nucleic Acids Res. 42, 320–324. https://doi.org/10.1093/nar/gku316.
14. Abramson, J., Adler, J., Dunger, J., Evans, R., Green, T., Pritzel, A., Ronneberger, O., Willmore, L., Ballard, A.J., Bambrick, J., et al. (2024). Accurate structure prediction of biomolecular interactions with AlphaFold 3. Nature 630, 493–500. https://doi.org/10.1038/s41586-024-07487-w.
