# Supplementary material for: Cetacean coronavirus spikes highlight S glycoprotein structural plasticity
Source: PLoS Pathog. 2026 Apr 8;22(4):e1013855. doi: 10.1371/journal.ppat.1013855 (PMC13061240; doi:10.1371/journal.ppat.1013855)
Supplement: Attachment — Submitted filename: Response to Reviewers.docx [file ppat.1013855.s006.docx]

**Response to Reviewers**

**Reviewers' Comments:**

Reviewer's Responses to Questions

***Part I - Summary*** *Please use this section to discuss strengths/weaknesses of study, novelty/significance, general execution and scholarship.*

**Reviewer #1:** In this manuscript, Hulswit et al. report high-resolution cryo-EM structures of two cetacean coronavirus spike proteins, showing that these spike trimers adopt a unique quaternary architecture, different from that of other coronaviruses. They have upright oriented S1^B^ domains covered by S1^A^ domains, and also a novel N-terminal S1^0^ domain that resembles a cupin fold. Glycoproteomic analysis has identified a mucin-like loop at the trimer apex with extensive N-glycosylation and O-glycosylation contributing to the dense glycan shield that map help evade host immune responses. The authors suggest that these interesting structural features offer new insights into the evolutionary mechanisms for coronaviruses to expand their host range.

The manuscript is very well written and all the experiments are relatively straightforward and technically sound. These new structures indeed help advance our understanding of structure-function of diverse coronavirus spike proteins.

**Reviewer #2**: In this study, the authors determined high‑resolution cryo‑EM structures of spike glycoproteins from two cetacean gammacoronaviruses that infect marine mammals (beluga whale and bottlenose dolphin). Their structural analyses revealed an alternative quaternary assembly in which the S1^B^ domains are tilted upright but shielded by extended loops from the S1^A^ domain and a mucin-like apex, effectively concealing the conserved receptor-binding motifs. They further identified a novel ~200‑residue N‑terminal domain (S1^0^) of host origin, structurally related to cupin proteins. Complementary glycoproteomic profiling showed that these spikes are heavily decorated with N‑glycans (>100 per trimer) and O‑glycans within the mucin‑like loop, forming a dense glycan shield. Together, these results demonstrate that cetacean coronaviruses have evolved unique structural and glycosylation features that expand the known diversity of coronavirus spikes, providing new insights into how these viruses adapt to novel hosts.

This is a well-written and straightforward manuscript that describes new insights into the diversity of coronavirus spike protein structure and architecture. The structural studies are performed to a high standard, and the analyses are thorough and thoughtful. Below are some minor comments and suggestions that may improve the manuscript.

***Part II – Major Issues: Key Experiments Required for Acceptance*** *Please use this section to detail the key new experiments or modifications of existing experiments that should be absolutely required to validate study conclusions.*

Generally, there should be no more than 3 such required experiments or major modifications for a "Major Revision" recommendation. If more than 3 experiments are necessary to validate the study conclusions, then you are encouraged to recommend "Reject".

Reviewer #1: (No Response)

Reviewer #2: No major issues

***Part III – Minor Issues: Editorial and Data Presentation Modifications*** *Please use this section for editorial suggestions as well as relatively minor modifications of existing data that would enhance clarity.*

**Reviewer #1**: There are only a few minor issues that should be addressed.
1. Discussion on the similarity between the S1^0^ domain and cupin fold is not very convincing, as they do not look like having a same fold, at least, as shown in Fig.3d (perhaps a better view would help?). The best matches are of bacterial origin and show only 8-9% sequence identity (not sure whether there are any conserved motifs critical for the cupin fold). So trying to conclude that “the S1^0^ sequence originates from either a mammalian or avian host given the observed species tropism of coronaviruses” sounds a bit stretchy.

We thank the reviewer for raising important points regarding the confidence in the proposed structural relationship between the CeCoV S1^0^ domain and members of the cupin superfamily. We agree that the structural similarity is difficult to interpret, particularly given that the top FoldSeek matches are predominantly bacterial and exhibit very low sequence identity (8–9%). As the reviewer notes, such a provenance would not readily align with the known host range of coronaviruses. In light of this, we have revised the manuscript text (lines 211–214) to more clearly reflect the uncertainty surrounding the evolutionary origin of this domain.

*“The top FoldSeek hits are predominantly of bacterial origin (Supplementary Data 1–2), an observation that is difficult to reconcile with the mammalian and avian host range of coronaviruses. At present, the evolutionary route by which this protein sequence entered a coronavirus genome therefore remains unclear.”*

At the same time, we note that both independent S1^0^ domains (from beluga whale and bottlenose dolphin, which share 46.5% amino-acid identity) yield consistent structural matches to cupin-like proteins using two distinct structure-based methods, FoldSeek and DALI. Although these relationships are likely distant, the convergence of results across both S1^0^ domains and both algorithms suggests that the similarity is not merely incidental. We therefore continue to interpret the S1^0^ domains as exhibiting some degree of structural relatedness to cupin-like folds, while being cautious about inferring their evolutionary origin.

Finally, in response to the reviewer’s suggestion, we attempted several alternative viewing angles for Fig. 3d to improve the visualization of the superposition. However, due to the apparent distance of the structural relationship, these adjustments did not yield a meaningfully clearer representation. We have therefore retained the current figure while clarifying the limitations in the text.

2. In the figure legend “Fig 1 SPA structure determination of BwCoV SED at 2.27 Å and BdCoV SED at 2.65 Å”, it seems to be the only place where SPA is mentioned and it should be spelt out (single particle analysis?) for general readers.

We thank the reviewer for pointing this out. To improve clarity for general readers, we have now spelled out the acronym upon first use in the figure legend. The revised title reads:

“Structure determination of BwCoV SED at 2.27 Å and BdCoV SED at 2.65 Å by single-particle analysis.”

3. The sentences –“*Neither hetero or local refinement appreciably improved the S1^0^ density, presumably due to intrinsic local protein flexibility and abundance of (N- and O-) glycosylation sites on the surface of CeCoV S1^0^ (described in detail later on) causing several S1^0^ regions to be absent in the final atomic models (see Table S2 for a complete overview including unresolved S1^A^ regions). The lack of observed conformational heterogeneity of domain S1^0^ relative to the rest of the spike protein complex underlines the existence of a singular S1^0^ state, packed against the S1^A^ domain by a modest network of hydrogen bonds involving main and side chain atoms (Fig. 3c)”* are a bit confusing for general readers. First, the S1^0^ density could not be improved because of protein flexibility, and then, “lack of observed conformational heterogeneity of domain S1^0^”. What they meant is probably that S1^0^ domain is rigid relative to the trimer, but it has some disordered loops.

We thank the reviewer for this helpful clarification. We agree that the original wording was confusing and did not clearly distinguish between local disorder within S1^0^ and the overall rigidity of the domain relative to the trimer. We have therefore rewritten the relevant passage (lines 180–187) to make this distinction explicit. The revised text now reads:

*“Neither hetero nor local refinement appreciably improved the S1^0^ density, presumably due to the presence of disordered loops and multiple (N- and O-) glycosylation sites on the surface of CeCoV S1^0^ (described in detail later on), which resulted in certain S1^0^ regions being unmodelled in the atomic models (see Table S2 for a complete overview, including unresolved S1^A^ regions). At the same time, because we did not detect conformational heterogeneity across particles, the S1^0^ domain appears rigid relative to the trimer as a whole and is positioned against the S1^A^ domain through a modest network of hydrogen bonds (Fig. 3c).”*

**Reviewer #2**: 
1. The clashscores for the structures are a little high, particularly for the higher-resolution BwCoV S model (21.00). The authors may try to loosen the geometric constraints during refinement to improve the score.

We thank the reviewer for this helpful suggestion. In response, we relaxed the geometric restraints during refinement, which reduced the clashscore of the BwCoV S model from 21.00 to 14.5, bringing it more in line with other reported coronavirus spike structures. The updated model has been deposited in the PDB accordingly.

2. The figures are, in general, clear and nicely illustrate the key points being made in the text. However, I feel Fig 1a could be improved. At a minimum, the EM maps could be removed so that readers can focus on the architecture of the spike. Improved coloring or surface representation may also help.

We thank the reviewer for this constructive suggestion. Following the recommendation, we have revised Fig. 1a by removing the EM maps and replacing the previous depiction with surface representations of the two atomic models presented in the manuscript. This adjustment improves visual clarity and better highlights the overall spike architecture. The figure legend has been updated accordingly.

3. line 24. The zero in S1^0^ needs to be superscripted.

Thanks for pointing this out, we have changed it accordingly.

4. Something seems off in lines 257-258: “Glycan densities were visible in the EM reconstructions for 26/37 N-unresolved regions of the maps.”

We thank the reviewer for pointing this out. In the revised manuscript this sentence now reads the following:

“*Glycan densities were visible in the EM reconstructions for 26/37 N- glycans on BwCoV and 29/35 on BdCoV S (Fig. 4b), with missing densities typically mapping to unresolved regions of the maps.*”

5. Out of curiosity, were the 2P stabilising mutations necessary? Beneficial?

We appreciate the interest of the reviewer and have wondered the same thing, but as we started from a 2P construct (gene was ordered as such) we do not know at this time whether these mutations are indeed required to obtain a structure or whether they have a significant stabilizing effect on the CeCoV S proteins. However, we anticipate such a stabilizing effect considering the relative conservation of the S2 subunit among coronavirus spike proteins where these mutations reside.

PLOS authors have the option to publish the peer review history of their article ([what does this mean?](https://eur03.safelinks.protection.outlook.com/?url=https%3A%2F%2Ftrack.editorialmanager.com%2FCL0%2Fhttps%3A%252F%252Fjournals.plos.org%252Fplospathogens%252Fs%252Feditorial-and-peer-review-process%2523loc-peer-review-history%2F1%2F010f019a968e4290-3d45280e-f8f7-49ae-9516-af177bed71a7-000000%2FK5H7svkPtOkL86ZcnCr_3ycziYdvY6WfpFbTLqWkNN8%3D237&data=05%7C02%7Cr.j.g.hulswit%40uu.nl%7Ca62faed57efe4c7cb62308de268f0d9f%7Cd72758a0a4464e0fa0aa4bf95a4a10e7%7C0%7C0%7C638990593036571149%7CUnknown%7CTWFpbGZsb3d8eyJFbXB0eU1hcGkiOnRydWUsIlYiOiIwLjAuMDAwMCIsIlAiOiJXaW4zMiIsIkFOIjoiTWFpbCIsIldUIjoyfQ%3D%3D%7C0%7C%7C%7C&sdata=2Rqe7iU5yjk5cHwX%2FmEjP3txQ8HuOeUf4mefTNXyZws%3D&reserved=0)). If published, this will include your full peer review and any attached files.

If you choose “no”, your identity will remain anonymous but your review may still be made public.

**Do you want your identity to be public for this peer review?** For information about this choice, including consent withdrawal, please see our [Privacy Policy](https://eur03.safelinks.protection.outlook.com/?url=https%3A%2F%2Ftrack.editorialmanager.com%2FCL0%2Fhttps%3A%252F%252Fwww.plos.org%252Fprivacy-policy%2F1%2F010f019a968e4290-3d45280e-f8f7-49ae-9516-af177bed71a7-000000%2FVHczMrkXSB01twiOGJF8LbJvBm6dxzwFdIKDNLJvAuQ%3D237&data=05%7C02%7Cr.j.g.hulswit%40uu.nl%7Ca62faed57efe4c7cb62308de268f0d9f%7Cd72758a0a4464e0fa0aa4bf95a4a10e7%7C0%7C0%7C638990593036607202%7CUnknown%7CTWFpbGZsb3d8eyJFbXB0eU1hcGkiOnRydWUsIlYiOiIwLjAuMDAwMCIsIlAiOiJXaW4zMiIsIkFOIjoiTWFpbCIsIldUIjoyfQ%3D%3D%7C0%7C%7C%7C&sdata=8xWvGSrPtMPZxYpBoWJmirQ83FHcK3OVR91fFXWQtcQ%3D&reserved=0).

Reviewer #1: No

Reviewer #2: No
